# Emergence and Regional Circulation of *Streptococcus iniae* Associated With Streptococcosis in Marine Cage‐Farmed Golden Rabbitfish (*Siganus guttatus*)

**DOI:** 10.1155/tbed/2656593

**Published:** 2026-07-13

**Authors:** Truong Dinh Hoai, Dang Thi Hoa, Nguyen Cong Thiet, Nguyen Thi Huong Giang, Doan Thi Nhinh, Tran Thi Nang Thu, Kim Van Van

**Affiliations:** ^1^ Faculty of Fisheries, Vietnam National University of Agriculture, Hanoi, Vietnam, vnua.edu.vn; ^2^ Center of Excellence and Innovation, Vietnam National University of Agriculture, Hanoi, Vietnam, vnua.edu.vn; ^3^ Faculty of Veterinary Medicine, Vietnam National University of Agriculture, Hanoi, Vietnam, vnua.edu.vn

**Keywords:** antimicrobial resistance, golden rabbitfish, marine cage aquaculture, *Streptococcus iniae*, virulence genes

## Abstract

Streptococcosis caused by *Streptococcus iniae* is an emerging bacterial disease affecting marine aquaculture worldwide. In recent years, recurrent outbreaks have been observed in golden rabbitfish (*Siganus guttatus*) cultured in marine cages across the northern and central coastal provinces of Vietnam. This study investigated the epidemiology, pathogenicity, virulence gene profiles and antimicrobial susceptibility of *S. iniae* associated with disease outbreaks in rabbitfish aquaculture. Between 2023 and 2025, streptococcosis outbreaks were documented across 46 marine cage farms in multiple coastal provinces, with cumulative mortality ranging from 10%–45%, particularly during warm‐water periods (28–33°C). Bacteriological examination consistently yielded *S. iniae*, which was confirmed by biochemical tests and species‐specific PCR assays. An experimental challenge demonstrated the high virulence of a representative isolate, with a median lethal dose (LD_50_) of 6.8 × 10^3^ CFU/fish. Histopathological examination revealed severe multi‐organ lesions, including prominent involvement of the brain, kidney, liver, spleen, gills and intestine, consistent with systemic bacterial infection. Molecular screening of virulence‐associated genes identified six distinct gene profiles among isolates, indicating substantial genetic heterogeneity within the circulating pathogen population. Several genes, including *pdi*, *simA*, *cpsB*, *cpsC*, *cpsD*, *pgm*, *hasA* and *ydfG* were detected in all isolates (100%), whereas others showed marked variability, with prevalence ranging from approximately 17% to 89% among isolates. Antimicrobial susceptibility testing revealed heterogeneous resistance patterns, with high resistance to neomycin (NE) and moderate resistance to trimethoprim–sulfamethoxazole (TRS), while most isolates remained susceptible to tetracyclines (TEs) and β‐lactam antibiotics. The widespread occurrence of outbreaks across multiple provinces and over consecutive years suggests sustained circulation of *S. iniae* within interconnected marine cage aquaculture systems. These findings provide new epidemiological insights into an emerging streptococcal disease affecting rabbitfish aquaculture and highlight the need for improved surveillance and disease management strategies in rapidly expanding marine cage‐farming sectors.

## 1. Introduction

The rapid expansion of marine aquaculture in tropical and subtropical regions has heightened concerns about emerging diseases affecting newly cultured fish species. Among these, the golden rabbitfish (*Siganus guttatus*) has attracted growing attention as a promising candidate for coastal aquaculture due to its high market value, favourable nutritional quality and adaptability to a wide range of environmental conditions [1–4]. The species is herbivorous, euryhaline and capable of thriving in both brackish water and nearshore marine environments where natural food resources are abundant [5]. In addition, *S. guttatus* shows good acceptance of formulated feeds and can be cultured at relatively high stocking densities, making it increasingly attractive for commercial production [6–8]. Consequently, farming of golden rabbitfish has expanded in many countries in recent years [1, 3, 5, 9]. Nevertheless, increasing production intensity and the wider adoption of marine cage culture have heightened the risk of infectious disease outbreaks, which now represent a significant challenge for sustainable rabbitfish production.

Among the bacterial diseases affecting cultured fish, streptococcosis remains a major health concern in both freshwater and marine aquaculture. *Streptococcus iniae* is recognised as one of the principal aetiological agents of this disease and has been associated with severe outbreaks across a variety of farmed fish species. Clinical manifestations commonly include abnormal swimming behaviour, neurological disorders, haemorrhages, septicaemia and extensive tissue damage in multiple organs [10–12]. Disease expression is often exacerbated by environmental and husbandry‐related stressors, particularly elevated water temperatures, which may increase host susceptibility and favour pathogen proliferation [13–16]. As a result, streptococcosis frequently leads to high cumulative mortality and considerable economic losses in intensive aquaculture systems.

The geographical and host distribution of *S. iniae* is remarkably broad, encompassing numerous cultured and wild fish species inhabiting freshwater, brackish‐water and marine environments. The pathogen has been documented in several economically important aquaculture species, such as rainbow trout, sturgeon, Nile tilapia, hybrid striped bass, barramundi, pompano and spotbanded scat [10, 15, 17–23]. In siganid fishes, however, available evidence remains limited and largely sporadic, being restricted to an outbreak in *Siganus canaliculatus* and the isolation of *S. iniae* from wild spinefoot (*Siganus rivulatus*) collected near marine cage farms [24, 25], as well as the detection of a single streptococcal isolate from golden rabbitfish (*Siganus guttatus*) [26]. These reports do not provide population‐level insight into the genetic variability, pathogenic attributes or antimicrobial resistance patterns of *S. iniae* infecting siganid hosts. Consequently, although *S. iniae* is recognised as an important pathogen in marine aquaculture, its occurrence, transmission dynamics and disease characteristics in *S. guttatus* remain insufficiently understood.

In many coastal farming areas, golden rabbitfish are often collected from wild sources and subsequently introduced into marine cage‐farming systems, frequently in proximity to other cultured fish species [1, 27]. This practice, together with the increasing prevalence of multi‐species farming, may promote interspecies transmission and long‐term maintenance of bacterial pathogens within aquaculture environments [25]. Under such conditions, rabbitfish may potentially function as reservoir or bridge hosts, contributing to the circulation, diversification and possibly the selection of more virulent or antimicrobial‐resistant *S. iniae* lineages. However, empirical data addressing these possibilities in the context of *S. guttatus* aquaculture remain scarce.

Therefore, the present study aimed to isolate and characterise *Streptococcus iniae* from diseased golden rabbitfish (*Siganus guttatus*) cultured in marine cage systems to investigate the epidemiological characteristics of the outbreaks, evaluate the pathogenicity of representative isolates, determine the distribution of virulence‐associated genes and assess antimicrobial susceptibility patterns in order to support disease management in marine aquaculture systems.

## 2. Materials and Methods

### 2.1. Fish Sample Collection

From 2023–2025, disease outbreaks in golden rabbitfish were investigated at 46 farms along the northern (Quang Ninh ‐ [QN] and Hai Phong [HP]) and central coastal provinces (Hue [H]) of Vietnam during the summer season (Figure S1). All surveyed farms operated marine cage‐culture systems. The stocking density in the sampled cages ranged from 28 to 60 fish/m^3^, with lower densities generally observed in cages containing larger fish due to routine grading and density reduction during grow‐out (Table 1). During outbreak investigations, six to 10 moribund fish were sampled from each affected farm. This approach ensured that representative clinical cases were obtained from outbreaks across geographically dispersed farms. Fish exhibiting clinical signs consistent with streptococcosis, including abnormal surface swimming, impaired balance, exophthalmia and periocular haemorrhages (Video S1), were preferentially selected for examination. A total of 392 golden rabbitfish were included in the laboratory investigations. Fish collected from nearby farms were transported live in oxygen‐filled plastic bags, while those from more distant locations were transported in sterile bags under chilled conditions (~4°C).

**Table 1:** Golden rabbitfish (*Siganus guttatus*) disease outbreaks and representative isolates.

Farm code	Average fish size (g)	Mortality rate (%)^a^	Number of fish collected	Water temperature (°C)^b^	Average culture densities in sample cages (Fish/m^3^)^c^	Representative isolates	Isolated year
QNDH1	25–30	20	7	28.3	44	SIQNHH1‐01	2025
QNDH2	40–45	15	7	28.2	48	SIQNHH2‐03	2025
QNDH3	45–50	20	10	29.3	43	SIQNDH3‐04	2025
QNDH4	53–60	20	10	29.2	45	SIQNDH4‐02	2025
QNDH5	60–65	20	10	28.4	52	SIQNDH5‐02	2025
QNVD6	65–68	30	8	29.7	60	**SIQNVD6-07** ^d^	2025
QNVD7	70–100	30	9	29.4	45	SIQNVD7‐03	2025
QNVD8	80–100	35	7	30.1	36	SIQNVD8‐06	2025
QNVD9	100–105	20	10	28.5	38	SIQNVD9‐09	2025
QNVD10	15–100	25	9	30.2	43	SIQNVD10‐04	2025
QNVD11	125–130	30	10	32.2	28	SIQNVD11‐02	2025
QNVD12	55–60	20	7	29.4	48	SIQNVD12‐07	2025
QNVD13	150–180	25	10	30.3	28	SIQNVD13‐10	2025
QNVD14	170–175	28	8	30.2	33	SIQNVD14‐05	2025
QNVD15	190–210	40	7	33.4	35	SIQNVD15‐06	2025
QNVD16	45–135	35	9	30.1	43	SIQNVD16‐03	2025
QNVD17	115–145	40	7	32.2	35	SIQNVD17‐05	2025
QNVD18	155–160	30	10	31.4	28	SIQNVD18‐02	2025
QNVD19	215–260	25	8	29.3	32	SIQNVD19‐04	2025
QNCP20	65–115	20	9	29.1	46	SIQNCP20‐08	2025
QNCP21	160–165	25	10	31.2	33	SIQNCP21‐03	2025
QNCP22	180–200	20	10	29.8	32	SIQNCP22‐05	2025
QNCP23	115–140	30	10	32.3	48	SIQNCP23‐06	2025
QNCP24	55–160	25	10	30.1	45	SIQNCP24‐07	2025
QNCP25	180–215	20	10	30.4	32	SIQNCP25‐05	2024
QNCP26	125–130	21	10	29.8	38	SIQNCP26‐01	2024
QNCP27	55–60	15	10	29.4	42	SIQNCP27‐04	2024
QNCP28	190–240	25	10	30.4	32	SIQNCP28‐03	2024
QNCP29	115–120	21	10	29.2	38	SIQNCP29‐06	2024
HPCB1	35–40	10	7	28.5	55	SIHPCB1‐03	2025
HPCB2	80–100	15	7	28.7	45	SIHPCB2‐04	2025
HPCB3	130–140	10	8	30.1	38	SIHPCB3‐01	2025
HPCB4	80–100	10	8	30.0	45	SIHPCB4‐08	2025
HPCB5	120–140	10	9	31.2	37	SIHPCB5‐06	2025
HPBB6	150–170	30	10	30.4	34	SIHPBB6‐04	2024
HPBB7	85–120	15	10	28.9	38	SIHPBB7‐02	2024
HPBB8	130–160	20	7	29.7	36	SIHPBB8‐03	2024
HPBB9	40–55	25	7	31.5	55	SIHPBB9‐01	2024
HPBB10	50–70	20	6	30.5	48	SIHPBB10‐03	2024
HPBB11	90–100	25	8	32.2	38	SIHPBB11‐04	2024
HPGL1	30–40	45	10	31.2	54	SIHPGL1‐01	2023
HPGL2	60–70	20	6	28.9	43	SIHPGL2‐03	2023
HPGL3	90–100	40	9	31.2	32	SIHPGL3‐02	2023
HTG4	45–50	25	9	30.5	43	SIHTG4‐03	2023
HTG5	65–70	40	8	30.4	40	SIHTG5‐06	2023
HTG6	75–85	25	7	31.3	42	SIHTG6‐04	2023

*Note:* Bold formatting and superscript “d” indicate the isolate selected for the experimental challenge.

^a^Cumulative mortality was estimated by the farm owner during the outbreak period.

^b^Water temperature was measured at the time of sampling.

^c^Stocking density refers to the average density in the sampled cages.

### 2.2. Microscopic Examination and Bacterial Isolation

To prioritise recovery of the predominant bacterial agents associated with disease outbreaks, gross examination and bacteriological culture were performed immediately upon receipt of fish samples. Gross pathological changes were recorded, and aseptically collected samples of kidney, brain and liver tissues were processed for bacteriological examination. Tissue samples were inoculated onto TSA (Merck, Darmstadt, Germany) supplemented with 2% (w/v) NaCl and cultured at 28°C for 48 h. After incubation, bacterial growth was examined, and the predominant colony morphotype, characterised by small, round, opaque and non‐pigmented colonies, was selected for purification. Purified isolates were preserved in tryptic soy broth (TSB) containing 20% (*v*/*v*) glycerol at −80°C for long‐term storage. To ensure broad geographic and farm‐level representation while avoiding redundant sampling of clonally similar isolates, one representative isolate per farm was retained for subsequent analyses.

### 2.3. Phenotypic Characterisation of Representative Bacterial Isolates

Phenotypic characterisation was undertaken as an initial screening step to assess the general taxonomic features of the recovered isolates prior to molecular confirmation. Colony appearance on TSA, cellular morphology following Gram staining and motility were first examined using standard microbiological methods. Enzymatic activities were then evaluated using oxidase and catalase tests, with interpretation based on the criteria described in Bergey’s Manual of Systematic Bacteriology [28]. Additional biochemical characterisation was performed using API 20 Strep strips (bioMérieux, France) following the manufacturer’s instructions. Isolates showing biochemical characteristics compatible with those of *Streptococcus iniae* ATCC 29178 were subjected to molecular confirmation and subsequent characterisation.

### 2.4. DNA Extraction and PCR Assays

For molecular confirmation, representative isolates grown on TSA containing 2% NaCl at 28°C for 48 h were used for genomic DNA extraction. A single purified colony from each culture was subjected to DNA extraction using the InstaGene Matrix kit (Bio‐Rad, California, USA), following the manufacturer’s protocol. The resulting DNA extracts were stored at −20°C prior to use as templates for PCR amplification.

To verify the species identity, two independent genetic markers of *Streptococcus iniae* were targeted, namely, the 16S rRNA gene and the lactate oxidase (*lctO*) gene. Species‐specific PCR targeting the 16S rRNA gene was performed using the primer pair Sin1/Sin2 (Sin1‐F: 5′‐CTAGAGTACACATGTAGCTAAG‐3′ and Sin2‐R: 5′‐GGATTTTCCACTCCCATTAC‐3′), which produces an amplicon of approximately 300 bp [25]. Confirmation was further supported by amplification of the *lctO* gene using primers Lox‐1/Lox‐2 (Lox‐1‐F: 5′‐AAGGGGAAATCGCAAGTGCC‐3′ and Lox‐2‐R: 5′‐ATATCTGATTGGGCCGTCTAA‐3′), generating a product of approximately 870 bp [29].

Each PCR assay was performed in a final volume of 25 μL consisting of the GoTaq Green Master Mix (Promega, USA), the appropriate primer pair, and genomic DNA obtained from a purified bacterial isolate. The PCR programme began with initial denaturation at 94°C for 5 min. This was followed by 35 cycles consisting of denaturation at 94°C for 1 min, target‐specific annealing at 55°C for the 16S rRNA assay or 58°C for the lctO assay for 1 min and extension at 72°C for 1 min. Amplification was completed by a final extension at 72°C for 5 min [25, 29]. Amplified products were analysed by agarose gel electrophoresis (1.5%) following staining with RedSafe nucleic acid stain (Intron, Korea). Bands were visualised and recorded using a gel documentation system (Bio‐Rad, USA). DNA extracted from *S. iniae* ATCC 29178 served as the positive control for all amplification reactions.

### 2.5. Challenge Experiments

To evaluate the pathogenic potential of field isolates, an experimental infection model was established using the golden rabbitfish (*Siganus guttatus*). A representative isolate (SIQNVD6−07) was selected for challenge experiments. The selected isolate was propagated in TSB at 28°C for 48 h, after which bacterial cells were recovered by centrifugation and suspended in sterile PBS. Tenfold serial dilutions were prepared to obtain bacterial suspensions containing approximately 1 × 10^3^ to 1 × 10^8^ CFU/mL.

Healthy golden rabbitfish (mean body weight ≈ 35 g) were sourced from a commercial marine farm in central Vietnam and maintained under laboratory conditions for a 7‐day acclimation period before the challenge. During the acclimation period, fish were monitored daily and showed no external lesions, abnormal swimming behaviour, loss of appetite or other clinical signs of disease. Prior to experimental infection, 10 fish were randomly sampled and screened bacteriologically to verify that no *S. iniae* or other clinically significant bacterial pathogens were detected. Following acclimation, fish were randomly assigned to seven experimental groups (15 fish per 250‐L tank containing 200 L of aerated seawater, three replicate tanks per treatment). Each fish received an intraperitoneal injection of 100 μL of the designated bacterial suspension. Control fish were injected with an equivalent volume of sterile PBS. Fish were maintained for 14 days under continuous aeration. Experimental fish were maintained under stable environmental conditions, including water temperatures of 29.5–31.0°C, salinity of 28–30‰, dissolved oxygen concentrations exceeding 5 mg/L, pH values of 7.0–7.5, and total ammonia nitrogen levels below 2 mg/L. Fish were inspected daily for abnormal behaviour, disease signs and mortality. Moribund or freshly dead fish were subjected to post‐mortem examination. To verify the identity of the challenge agent, bacteria were re‐isolated from the brain, liver and kidney of experimentally infected fish and confirmed as *S. iniae* by biochemical testing and PCR analysis. The LD_50_ value was estimated according to the method described previously [30].

### 2.6. Histopathological Examination

Histopathological examination was conducted using tissues collected from naturally infected fish (*n* = 5), experimentally challenged moribund fish (*n* = 30) and healthy control fish (*n* = 5). Liver, kidney, spleen, gill, brain and intestinal tissues were fixed in 10% neutral‐buffered formalin for 24 h and processed using routine histological techniques. Following dehydration and paraffin embedding, tissue sections (5 μm) were prepared and stained with haematoxylin and eosin (H&E). Microscopic lesions were evaluated using an Olympus imaging system (Tokyo, Japan). Representative pathological changes were photographed and compared across the study groups.

### 2.7. Detection of Virulence‐Associated Genes

Virulence‐associated genes were screened by PCR to assess the distribution of putative virulence determinants among the *S. iniae* isolates. A total of 14 genes were selected based on previous reports [25, 31, 32]. The selected genes represented putative virulence determinants associated with adhesion, capsule production, toxin activity, and other pathogenic functions, including *scpI*, *pdi*, *sagA*, *simA*, *cpsB*, *cpsC*, *cpsD*, *cfi*, *pgm*, *tagU*, *nisF*, *ykpA*, *ydfG* and *hasA*. The selected targets were grouped into six multiplex PCR panels (Table 2).

**Table 2:** Primers used for multiplex PCR detection of *Streptococcus iniae* virulence‐associated genes.

PCR set	Primer	GenBank ID/accession number	Amplicon size (bp)	Primer sequence (5′–3′)	Putative function
Set 1 (4 genes)	*cpsC*‐F	gene0067	690	ATGAACACAAGCGAAAACA	Capsular polysaccharide
	*cpsC*‐R			TTACATTTTATTTGTGTTTGGA	
	*cpsD*‐F	gene0068/AY904444	534	TGGTGAAGGAAAGTCAACCAC	Capsular polysaccharide
	*cpsD*‐R			TCTCCGTAGGAACCGTAAGC	
	*sagA*‐F	AY904444	190	AGGAGGTAAGCGTTATGTTAC	Streptolysin S
	*sagA*‐R			AAGAAGTGAATTACTTTGG	
	*16S rRNA*‐F	—	300	CTAGAGTACACATGTAGCTAAG	—
	*16S rRNA*‐R			GGATTTTCCACTCCCATTAC	
Set 2 (4 genes)	*pdi*‐F	gene1804/FJ664396	381	TTTCGACGACAGCATGATTG	Polysaccharide deacetylase
	*pdi*‐R			GCTAGCAAGGCCTTCATTTG	
	*pgm*‐F	AY846302	490	TATTAGCTGCTCACGGCATC	Phosphoglucomutase
	*pgm*‐R			TTAGGGTCTGCTTTGGCTTG	
	*nisF*‐F	gene1900	699	ATGACTAACATCATTGAAACGA	β‐haemolysin
	*nisF*‐R			TCATACCCCTTCCTTCTTTA	
	*16S rRNA*‐F	—	300	CTAGAGTACACATGTAGCTAAG	—
	*16S rRNA*‐R			GGATTTTCCACTCCCATTAC	
Set 3 (3 genes)	*simA*‐F	gene0297/EU693238	994	AATTCGCTCAGCAGGTCTTG	M‐like protein
	*simA* ‐R			AACCATAACCGCGATAGCAC	
	*ydfG*‐F	gene0928	759	ATGCTTAAAAAAATTGCTCTT	β‐haemolysin
	*ydfG*‐R			TTAATCCCTATGAACCGGT	
	*16S rRNA*‐F	—	300	CTAGAGTACACATGTAGCTAAG	—
	*16S rRNA*‐R			GGATTTTCCACTCCCATTAC	
Set 4 (3 genes)	*cfi*‐F	gene0120	771	ATGAACTCTCAACACATTTTACG	CAMP factor
	*cfi*‐R			TTAGTTAAGAGCAGCTGTTAAGG	
	*tagU*‐F	gene0065	1464	ATGGCACATTCCAGAAGTAA	Capsular polysaccharide
	*tagU*‐R			TCATTTACTTTCCTCCATTACT	
	*16S rRNA*‐F	—	300	CTAGAGTACACATGTAGCTAAG	—
	*16S rRNA*‐R			GGATTTTCCACTCCCATTAC	
Set 5 (3 genes)	*scpI*‐F	gene0773/EU693239	822	GCAACGGGTTGTCAAAAATC	C5a peptidase
	*scpI*‐R			GAGCAAAAGGAGTTGCTTGG	
	*ykpA*‐F	gene0655	1620	TTGCTTACTGTTTCTGATGTG	β‐haemolysin
	*ykpA*‐R			TTATTTCCAAAGTTCTGCAA	
	*16S rRNA*‐F	—	300	CTAGAGTACACATGTAGCTAAG	—
	*16S rRNA*‐R			GGATTTTCCACTCCCATTAC	
Set 6 (3 genes)	*hasA*‐F	gene1372	1242	ATGGAAAAACTGAAAAATCTAA	Hyaluronic acid capsule
	*hasA*‐R			TTAAGTAAGAGGTTCTTCTTCCT	
	*cpsB*‐F	gene0066	732	ATGATTGACATCCATTCCCA	Capsular polysaccharide
	*cpsB*‐R			CTATAAATAATCATTTTCAATCAGG	
	*16S rRNA*‐F	—	300	CTAGAGTACACATGTAGCTAAG	—
	*16S rRNA*‐R			GGATTTTCCACTCCCATTAC	

PCR reagents and post‐amplification analyses were performed according to the procedures outlined in Section 2.4. Multiplex PCR amplification was performed under the cycling conditions described in Section 2.4, except that an annealing temperature of 58°C was used for all virulence‐gene assays. Gene detection patterns were used to evaluate virulence‐gene diversity and to categorise isolates into distinct virulence profiles.

### 2.8. Antimicrobial Susceptibility Test

The antimicrobial susceptibility of the *S. iniae* isolates was evaluated using the disk diffusion method. The interpretation of inhibition zones followed the Clinical and Laboratory Standards Institute (CLSI) recommendations for aquatic bacterial pathogens [33]. Where species‐specific breakpoints for *S. iniae* were unavailable, published interpretative criteria were applied [22].

A panel of 12 antimicrobial agents representing nine antibiotic classes was evaluated on Mueller–Hinton agar (Oxoid, UK), including doxycycline (DOX, 30 μg), tetracycline (TE, 30 μg), oxytetracycline (OTC, 30 μg), amoxicillin (AMO, 10 μg), oxacillin (OX, 1 μg), cefotaxime (CT, 30 μg), cefuroxime (Cu, 30 μg), AMO–clavulanic acid (AC, 20/10 μg), erythromycin (ERY, 15 μg), florfenicol (FLO, 30 μg), neomycin (NE, 30 μg) and trimethoprim–sulfamethoxazole (TRS, 1.25/23.75 μg). Bacterial inocula were standardised to the turbidity of a 0.5 McFarland suspension before being evenly distributed onto Mueller–Hinton agar plates using sterile cotton swabs. Antimicrobial disks were applied to the agar surface, and plates were subsequently incubated at 28°C for 48 h. Following incubation, inhibition zones were measured, and isolates were categorised as susceptible, intermediate or resistant according to the selected interpretive criteria.

### 2.9. Statistical Analysis

Epidemiological and experimental data were analysed using a combination of descriptive and inferential statistical approaches. Outbreak‐related variables, including fish size, water temperature, stocking density and cumulative mortality, were initially summarised using descriptive statistics. Spearman’s rank correlation analysis was performed to evaluate the relationships between cumulative mortality and potential outbreak‐associated factors, including stocking density and water temperature, at the farm level. The frequency of individual virulence genes and the distribution of antimicrobial susceptibility categories were summarised descriptively. For the experimental infection trial, differences in survival and mortality among challenge groups were compared using the chi‐square (*χ* ^2^) test, and time‐to‐mortality data were presented as Kaplan–Meier survival curves. Statistical analyses were conducted using the IBM SPSS Statistics version 27.0 (IBM Corp., Armonk, NY, USA).

## 3. Results

### 3.1. Clinical Signs, Gross Lesions, Bacterial Isolation and Identification

Across the 46 surveyed marine cage farms, disease outbreaks in golden rabbitfish were recorded mainly during periods of elevated water temperature (28.2–33.2°C), with cumulative mortalities ranging from approximately 10% to 45% within the first 7 days after disease onset (Table 1). At the time of sampling, affected fish spanned a wide size range, with body weights ranging from 15–260 g, indicating that outbreaks occurred across multiple production stages. Among the factors evaluated, water temperature was the variable most strongly associated with increased cumulative mortality (Spearman’s *ρ* = 0.614, *p* < 0.001), whereas no significant associations were detected between outbreak severity and stocking density in the present dataset.

**Figure 1:**
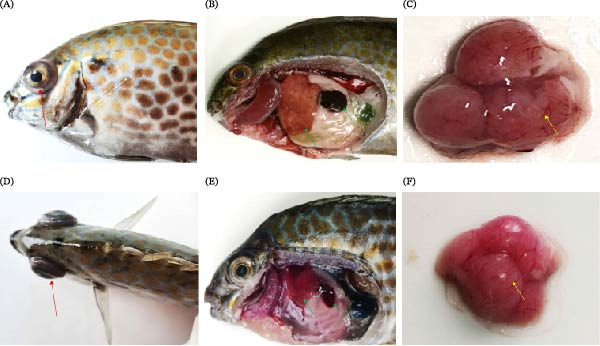
Clinical signs and gross pathological features of diseased golden rabbitfish (*Siganus guttatus*). (A–C) Gross appearance and lesions of naturally diseased fish showing exophthalmos (red arrow) and haemorrhagic lesions in internal organs (green arrows) and brain (yellow arrow). (D–F) Fish experimentally challenged with *Streptococcus iniae* isolate SIQNVD6‐07 showing clinical signs comparable to those observed in naturally infected fish.

Affected fish commonly showed abnormal swimming behaviour, including lethargy, loss of equilibrium and disoriented movement near the water surface (Video S1). External examination frequently revealed exophthalmia and periocular haemorrhages, whereas internal inspection consistently demonstrated congestion and multifocal haemorrhages in highly vascularised organs, particularly the brain, liver, kidney and spleen (Figure 1A–C).

**Figure 2:**
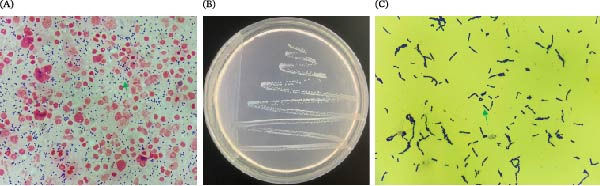
Microscopic and colony morphology of *Streptococcus iniae* isolated from diseased golden rabbitfish. (A) Gram‐stained smear showing abundant coccoid bacteria in the head kidney. (B) Colony morphology on TSA supplemented with 2% NaCl after 48 h incubation at 28°C. (C) Cellular morphology of *S. iniae* showing Gram‐positive cocci arranged in chains.

Gram‐stained smears prepared from the brain, kidney, liver and spleen of diseased fish consistently revealed abundant Gram‐positive coccoid bacteria, predominantly arranged in chains, indicating a heavy bacterial burden in affected tissues (Figure 2A). Consistent with these microscopic observations, bacteriological culture of kidney, brain and liver samples yielded a single dominant bacterial morphotype from all farms investigated. On tryptic soy agar supplemented with 2% NaCl, colonies were small, circular, opaque and non‐pigmented (Figure 2B). Microscopic examination of pure cultures confirmed Gram‐positive coccoid cells, typically arranged in short chains (Figure 2C).

A total of 46 representative isolates, each originating from a different outbreak farm, were selected for further analysis based on consistent clinical findings, Gram‐staining results and bacteriological culture. Conventional biochemical tests and API 20 Strep profiles showed characteristics consistent with those of the reference strain *Streptococcus iniae* ATCC 29178. None of the isolates exhibited motility, oxidase activity, catalase activity or hippurate hydrolysis. In contrast, positive reactions were consistently recorded for pyrrolidonyl arylamidase (PYRA) and arginine dihydrolase (ADH). The remaining biochemical characteristics evaluated, including β‐glucuronidase, Voges–Proskauer, α‐galactosidase and β‐galactosidase activities, were uniformly negative (Table 3).

**Table 3:** Morphological and biochemical characteristics of *Streptococcus iniae* isolates retrieved from diseased golden rabbitfish in this study.

Characteristic	Isolates in this study (*n* = 46)	Reference strain *S. iniae* ATCC 29178
Gram stain	+	+
Cell morphology	Cocci	Cocci
Oxidase	−	−
Catalase	−	−
Motility	−	−
Colony size	0.5–1 mm	0.5–1 mm
Colony colour (TSA^+^)	Milky white	Milky white
Voges Proskauer (VP)	−	−
Hippuric acid (HIP)	−	−
Esculin (ESC)	+	+
Pyrrolidonyl arylamidase (PYRA)	+	+
α‐Galactosidase (α ‐GAL)	−	−
β‐Glucuronidase (β‐GUR)	−	−
β‐Galactosidase (β‐GAL)	−	−
Alkaline phosphatase (PAL)	+	+
Leucine aminopeptidase (LAP)	+	+
Arginine dihydrolase (ADH)	+	+
Ribose (RIB)	+	+
Arabinose (ARE)	−	−
Mannitol (MAN)	+	+
Sorbitol (SOR)	−	−
Lactose (LAC)	−	−
Trehalose (TRE)	+	+
Inulin (INU)	−	−
Raffinose (RAF)	−	−
Amidon (AMD)	+	+
Glycogen (GLYG)	+	+

*Note:* (+) positive reaction; (−) negative reaction.

**Figure 3:**
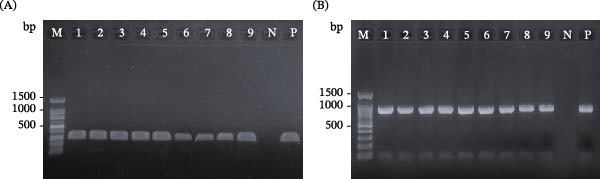
PCR identification of bacterial isolates using *S. iniae*‐specific primer pairs. (A) Sin1/Sin2. (B) Lox‐1/Lox‐2. M: DNA marker; lanes 1–9: representative bacterial isolates recovered from diseased golden rabbitfish; N: negative control (sterile physiological saline); and P: positive control (DNA of the reference strain *S. iniae* ATCC 29178).

PCR analysis targeting two independent genetic markers (16S rRNA and *lctO*) confirmed the identity of all representative isolates as *Streptococcus iniae*. Amplification products of the expected sizes (~300 and ~870 bp, respectively) were obtained from all 46 isolates (Figure 3).

### 3.2. Bacterial Challenge Experiments

**Figure 4:**
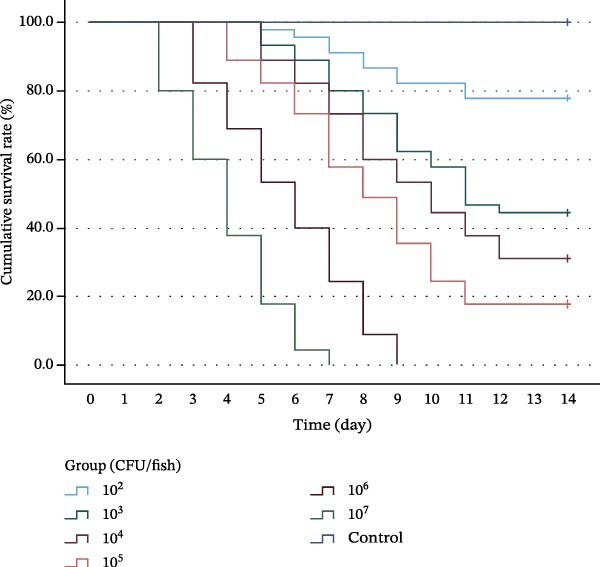
Kaplan–Meier survival curves of golden rabbitfish (*n =* 45 per treatment) following intraperitoneal challenge with *Streptococcus iniae* isolate SIQNVD6‐07 at different bacterial doses over a 14‐day observation period.

Challenge exposure to isolate SIQNVD6−07 produced a clear dose‐dependent mortality pattern throughout the 14‐day trial (Figure 4). Fish receiving higher inoculum concentrations (10^6^–10^7^ CFU/fish) developed clinical signs earlier and experienced substantially greater mortality than fish exposed to lower challenge doses. At 14 days post‐challenge, the cumulative mortality at 10^5^ CFU/fish was significantly higher than that at 10^3^ CFU/fish (*χ* ^2^ test, *p* < 0.05). Mortality in fish challenged with 10^4^ CFU/fish was intermediate and did not differ significantly from that observed in either the 10^3^ or 10^5^ CFU/fish groups (Figure S2). Fish receiving sterile PBS remained clinically normal throughout the experiment, and no mortality was observed in the control group. Experimentally infected fish developed clinical manifestations comparable to those observed during field outbreaks, including erratic swimming behaviour, exophthalmia and internal haemorrhagic lesions (Figure 1D–F), demonstrated successful reproduction of streptococcosis under controlled laboratory conditions. Analysis of cumulative mortality recorded over the 14‐day observation period yielded an estimated LD_50_ value of 6.8 × 10^3^ CFU/fish for isolate SIQNVD6−07, calculated using the Reed and Muench method [29]. *S. iniae* was consistently recovered from the brain, liver and kidney of moribund fish and subsequently confirmed by biochemical and molecular analyses, thereby satisfying Koch’s postulates.

### 3.3. Histopathological Examination

**Figure 5:**
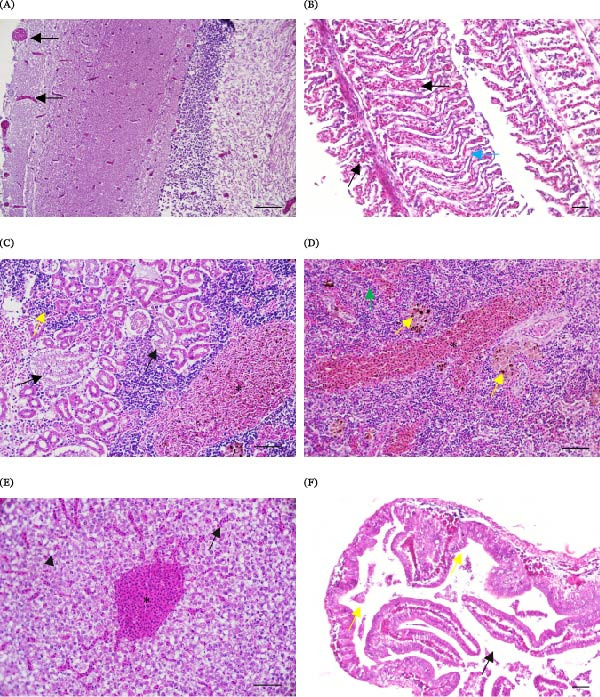
Histopathological alterations in golden rabbitfish naturally infected with *Streptococcus iniae*. (A) Brain showing vascular congestion and focal haemorrhages (black arrows). (B) Gills with disruption of filament architecture accompanied by haemorrhage and congestion (black arrow) and cellular oedema (blue arrow). (C) Kidney exhibiting degeneration and disorganisation of tubular and glomerular structures (black arrows), with focal haemorrhage and congestion ( ^∗^) and the presence of abundant coccoid bacteria (yellow arrow). (D) Spleen showing marked congestion ( ^∗^) and inflammatory changes consistent with splenitis (yellow arrows), with bacterial infiltration (green arrow). (E) Liver displaying severe haemorrhage (black arrow), vascular congestion ( ^∗^) and hepatocellular lipid accumulation (arrow head). (F) Intestine showing extensive epithelial necrosis (black arrows), inflammatory cell infiltration and haemorrhage (yellow arrows). Scale bars = 10 μm.

Histopathological analysis revealed extensive tissue alterations in golden rabbitfish, both naturally infected and experimentally challenged by *S. iniae*, involving multiple organ systems (Figure 5). Lesions were particularly prominent in highly vascularised tissues, where circulatory disturbances and inflammatory changes predominated. In the brain, marked vascular congestion and focal haemorrhages were frequently observed, consistent with severe involvement of the central nervous system. Gill tissues exhibited disruption of the filament architecture associated with oedema and haemorrhagic changes. In the kidney, the normal renal architecture was severely disrupted, with degeneration and disorganisation of tubular and glomerular structures accompanied by marked vascular congestion and focal haemorrhages. The spleen exhibited conspicuous inflammatory changes and congestion, consistent with splenitis. In the liver, pronounced sinusoidal dilatation and congestion of central veins were observed together with variable degrees of hepatocellular lipid accumulation, indicating combined haemodynamic and metabolic disturbance. The intestinal mucosa displayed extensive epithelial necrosis, infiltration of inflammatory cells and accumulation of cellular debris within the lumen.

### 3.4. Distribution of Virulence‐Associated Genes

Screening of the *S. iniae* isolates recovered from golden rabbitfish revealed a heterogeneous distribution of 14 virulence‐associated genes (Table 4). Several genes, including those involved in capsular polysaccharide synthesis and other key virulence determinants, were highly conserved among the isolates. All isolates (100%) harboured genes associated with M‐like protein (*simA*), polysaccharide deacetylation (*pdi*), phosphoglucomutase (*pgm*), capsular polysaccharide synthesis (*cpsB*, *cpsC* and *cpsD*) and β‐haemolysin (*ydfG*). In contrast, several genes showed variable presence, including those encoding C5a peptidase (*scpI*, 87.0%), CAMP factor (*cfi*, 89.1%), β‐haemolysin‐associated proteins (*nisF*, 84.8%; *ykpA*, 56.5%), streptolysin S (*sagA*, 82.6%) and capsular polysaccharide (*tagU*, 17.4%), indicating substantial genetic diversity within the *S. iniae* population.

**Table 4:** Percentage of *Streptococcus iniae* isolates harbouring virulence‐associated genes.

Virulence factors	C5a peptidase	M‐like protein	Polysaccharide deacetylase	Phospho‐glucomutase	CAMP factor	Capsular polysaccharide				β‐haemolysin			Hyaluronic acid capsule	Streptolysin, S
Gene	*scpI*	*simA*	*pdi*	*pgm*	*cfi*	*cpsB*	*cpsC*	*cpsD*	*tagU*	*nisF*	*ykpA*	*ydfG*	*hasA*	*sagA*
Percentage (%)	87.0	100.0	100.0	100.0	89.1	100.0	100.0	100.0	17.4	84.8	56.5	100.0	69.6	82.6

**Figure 6:**
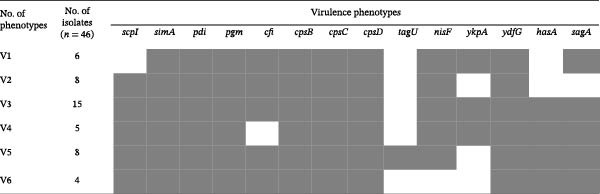
Virulence gene profiles of *Streptococcus iniae* isolated from golden rabbitfish (*Siganus guttatus*). Dark grey boxes indicate genes detected and uncoloured boxes denote genes absent.

**Figure 7:**
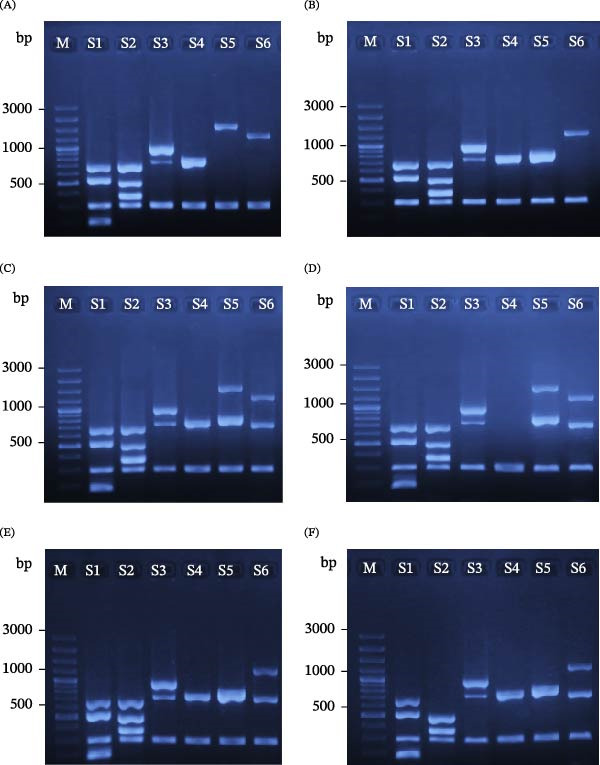
Agarose gel electrophoresis of multiplex PCR products representing six virulence gene phenotypes of *Streptococcus iniae* identified in this study. (A) Phenotype 1. (B) Phenotype 2. (C) Phenotype 3. (D) Phenotype 4. (E) Phenotype 5. (F) Phenotype 6. M: DNA marker; lanes S1–S6: six multiplex PCR sets targeting different virulence‐associated genes; internal positive control: 16S rRNA gene (300 bp).

Based on the combination of detected genes, the isolates could be classified into six distinct virulence profiles (V1–V6) (Figures 6 and 7). The number of genes per phenotype ranged from 9 to 13. Phenotype 3 was the most frequently observed, accounting for 15 of 46 isolates (32.6%). These results indicate the co‐circulation of multiple *S. iniae* lineages with distinct virulence gene complements in marine cage‐farmed golden rabbitfish.

### 3.5. Antibiotic Resistance

Antimicrobial susceptibility testing revealed heterogeneous resistance patterns among the *S. iniae* isolates across the tested antibiotic panel (Table 5). Overall, high levels of resistance to NE (69.6%) were observed, whereas moderate resistance was observed for TRS (30.4%). In contrast, most isolates remained susceptible to several commonly used antimicrobial agents, including TEs and β‐lactam antibiotics. These patterns suggest that although several first‐line antibiotics remain effective, resistance to specific compounds, such as NE, is already widespread in *S. iniae* from marine cage‐farmed golden rabbitfish.

**Table 5:** Antimicrobial susceptibility profiles of *Streptococcus iniae* isolates (*n* = 46) determined by disk diffusion.

Antibiotics	Antimicrobial susceptibility of *S. iniae* (Number of isolates [%])		
	*S*	*I*	*R*
Oxacillin (OX)	39 (84.8)	0 (0)	7 (15.2)
Amoxicillin (AMO)	39 (84.8)	7 (15.2)	0 (0)
Amox‐clavulanic (AC)	46 (100)	0 (0)	0 (0)
Cefotaxime (CT)	40 (87.0)	6 (13.0)	0 (0)
Cefuroxime (CU)	46 (100)	0 (0)	0 (0)
Trimethoprim/sulfamethoxazole (TRS)	25 (54.3)	7 (15.2)	14 (30.4)
Erythromycin (ERY)	25 (54.3)	14 (30.4)	7 (15.2)
Doxycycline (DOX)	46 (100)	0 (0)	0 (0)
Tetracycline (TE)	46 (100)	0 (0)	0 (0)
Oxytetracycline (OTC)	46 (100)	0 (0)	0 (0)
Florfenicol (FLO)	39 (84.8)	0 (0)	7 (15.2)
Neomycin (NE)	0 (0)	14 (30.4)	32 (69.6)

Abbreviations: *I*, intermediately susceptible; *R*, resistant; *S*, susceptible.

## 4. Discussion

This study provides the first comprehensive investigation of streptococcosis caused by *Streptococcus iniae* in marine cage–farmed golden rabbitfish (*Siganus guttatus*) across the northern and central coastal regions of Vietnam. Disease outbreaks were documented in 46 farms distributed across multiple provinces over three consecutive years, indicating that *S. iniae* is unlikely to represent isolated sporadic events and may be established within regional marine cage aquaculture systems. Experimental infection confirmed the high virulence of the representative isolate, while histopathological examination demonstrated severe systemic lesions consistent with septicaemic streptococcosis. Molecular screening further revealed substantial heterogeneity in virulence gene profiles among isolates, together with variable antimicrobial susceptibility patterns. These findings extend previous limited reports of *S. iniae* in siganid fishes and provide new epidemiological insights into the circulation of this pathogen in rabbitfish aquaculture. These observations complement our recent report of *S. iniae* outbreaks in marine cage‐cultured pompano (*Trachinotus* spp.) in Vietnam, suggesting that the pathogen is now established across multiple marine finfish production systems in the region [23]. Given the increasing expansion of marine cage farming and the frequent movement of fish and equipment between coastal farming areas, such conditions may facilitate the persistence and regional dissemination of bacterial pathogens within interconnected aquaculture networks [34, 35].

Previous reports of *S. iniae* in siganid fishes have been limited to isolated observations, including an outbreak in *Siganus canaliculatus* [24] and the detection of the bacterium in wild *Siganus rivulatus* near marine cage farms [25]. Evidence in *S. guttatus* has been even more limited, consisting primarily of the recovery of a single isolate without broader epidemiological investigation [26]. By analysing isolates obtained from 46 independent farms across multiple coastal provinces, the present study provides the first population‐scale assessment of *S. iniae* associated with golden rabbitfish aquaculture. The repeated occurrence of outbreaks across geographically separated sites suggests that the pathogen may be maintained within regional cage‐farming environments rather than representing sporadic introduction events.

Disease outbreaks were consistently observed during periods of elevated water temperature (~28–33°C) across multiple coastal provinces and over three consecutive years, in agreement with previous reports indicating that streptococcosis in fish is strongly temperature‐dependent [20, 34, 36]. This pattern was further supported by the correlation analysis, which identified water temperature as the variable most strongly associated with cumulative mortality, whereas no significant associations were detected between mortality and stocking density. Elevated temperature can enhance bacterial growth while simultaneously reducing immune competence in teleost fish, thereby increasing susceptibility to systemic infection [15, 34]. Although high stocking density is often considered a risk factor for streptococcosis in intensive aquaculture systems [13, 16], such an association was not evident in the present dataset. This may be attributable to the considerable variation in fish size and production stage among the sampled farms, which resulted in substantial differences in stocking density due to routine grading and density reduction during grow‐out. Furthermore, stocking density was recorded only at the time of sampling and may therefore not fully reflect density conditions throughout the outbreak period. Consequently, further studies involving a larger number of farms, broader geographic coverage and more detailed monitoring of stocking density throughout the production cycle are needed to better evaluate the role of stocking density in the occurrence and severity of streptococcosis outbreaks in golden rabbitfish aquaculture. In open marine cage systems, hydrodynamic connectivity between farms may further facilitate pathogen dispersal through water currents. Unlike land‐based aquaculture systems, marine cages remain continuously exposed to the surrounding environment, allowing potential exchange of microorganisms between cultured stocks and adjacent coastal waters [35]. The repeated occurrence of outbreaks across provinces and years, together with the detection of genetically diverse isolates across farming locations, is consistent with the possibility of the long‐term circulation of *S. iniae* within interconnected coastal aquaculture networks rather than a single short‐lived introduction event. Open marine cage systems create ecological interfaces where farmed fish, wild fish populations and environmental microbial communities interact continuously. Such connectivity may facilitate the persistence and regional dissemination of opportunistic bacterial pathogens, such as *S. iniae*, allowing pathogen populations to circulate among farms and surrounding aquatic environments. Under these conditions, marine cage aquaculture can function as a dynamic ecological network in which pathogens are maintained through repeated host–environment interactions rather than isolated farm‐level outbreaks.

Experimental infection demonstrated clear dose‐dependent mortality and reproduced the neurological and systemic signs characteristic of streptococcosis. Experimental infection demonstrated the substantial pathogenicity of the selected isolate, yielding an LD_50_ of 6.8 × 10^3^ CFU/fish, and reproduced the neurological and systemic manifestations observed under field conditions. The LD_50_ falls within the lower range reported for *S. iniae* infections in several cultured fish species, suggesting that the tested isolate has relatively high virulence [20, 36]. However, virulence in aquatic streptococci should not be considered a fixed intrinsic bacterial property determined solely by the gene presence. Disease expression reflects dynamic interactions among the bacterial genotype, host physiological status and environmental stressors [37]. Marine cage farming exposes fish to fluctuating temperature, high stocking density, handling procedures and variable water quality, all of which may suppress innate immune responses through stress‐mediated endocrine pathways [38]. Moreover, several streptococcal virulence mechanisms, including capsular polysaccharide synthesis and surface‐associated adhesins, are influenced by environmental conditions and regulatory pathways [39]. Consequently, the mortality observed in experimental challenge trials reflects host–pathogen interactions under controlled laboratory conditions and may not fully capture the environmental variability present under farm conditions.

Histopathological examination revealed severe systemic lesions affecting multiple organs, including the brain, kidney, liver, spleen, gills and intestine. Vascular congestion, haemorrhage and inflammatory infiltration were particularly evident in highly vascularised tissues. The prominent lesions observed in the brain are consistent with bacterial meningoencephalitis, a well‐recognised pathological feature of streptococcal infections in fish and a major factor underlying the neurological signs frequently observed in diseased animals [10, 34, 36]. The widespread tissue damage observed in this study supports the interpretation that streptococcosis in golden rabbitfish represents a systemic septicaemic disease involving multiple organ systems. The predominance of lesions in highly vascularised organs further supports the systemic dissemination of the bacterium through the circulatory system following infection.

Molecular screening revealed that several putative virulence determinants were detected in all isolates, including those encoding the M‐like protein (*simA*), the polysaccharide deacetylase (*pdi*), phosphoglucomutase (*pgm*) and the capsular polysaccharide synthesis proteins (*cpsB*, *cpsC* and *cpsD*). These determinants are considered important components of the virulence architecture of *S. iniae*. Capsule formation plays a critical role in resistance to phagocytosis, while surface proteins such as M‐like proteins contribute to host colonisation and immune evasion [25, 40, 41]. The universal presence of these determinants suggests a shared virulence backbone necessary for successful infection in *S. guttatus*. In contrast, accessory determinants, such as *scpI*, *cfi*, *nisF*, *ykpA*, *tagU*, *hasA* and *sagA*, showed variable distribution among isolates, resulting in six distinct virulence phenotypes. The coexistence of multiple phenotypes across geographically separated farms indicates that *S. iniae* populations circulating in marine cage aquaculture are not clonally uniform but instead display substantial genetic heterogeneity. Similar heterogeneity in virulence gene composition has also been reported among *S. iniae* isolates infecting other cultured fish species, including marine cage‐cultured pompano in Vietnam [12, 21, 23, 42]. Such heterogeneity may enhance the pathogen’s adaptive capacity to variable environmental conditions and host populations, potentially contributing to the long‐term persistence of streptococcosis within aquaculture ecosystems.

The representative isolate used for experimental infection (SIQNVD6‐07) belonged to the most frequently observed virulence phenotype (V3) and originated from the Quang Ninh–Van Don farming area, which accounted for the largest proportion of the surveyed farms. However, only a single isolate representing a single phenotype was evaluated experimentally, which limits the extrapolation of virulence characteristics to all circulating *S. iniae* populations. Functional differences among phenotypes, therefore, remain to be clarified. Comparative infection trials using representative isolates from each phenotype, conducted under strictly standardised environmental conditions, including identical temperature, salinity, stocking density and handling protocols, would be required to determine whether accessory gene variability translates into measurable differences in mortality kinetics, LD_50_ values, organ colonisation patterns or host immune responses. Such studies would help distinguish functional virulence variation from neutral genetic diversity within aquaculture‐associated populations.

Antimicrobial susceptibility testing revealed heterogeneous resistance patterns among the isolates. High resistance was observed for NE, whereas moderate resistance occurred for TRS. In contrast, most isolates remained susceptible to TEs and β‐lactam antibiotics. The emergence of antimicrobial resistance in aquaculture‐associated bacteria has often been associated with selective pressure from antibiotic use in fish farming and the environmental dissemination of antibiotic residues [43, 44]. In open marine cage environments, antimicrobial compounds and resistant bacteria may disperse through water currents, potentially influencing microbial communities beyond individual farms [45, 46]. Continued surveillance of antimicrobial susceptibility patterns and responsible antibiotic management are therefore essential to maintain treatment effectiveness and limit the spread of resistance determinants.

Golden rabbitfish used for aquaculture are often derived from wild‐caught juveniles and are commonly reared in coastal areas where multiple marine fish species are cultured in close proximity, such as pompano (*Trachinotus* spp.) and cobia (*Rachycentron canadum*) [1, 27]. Under such conditions, opportunities for pathogen exchange among host species are likely to occur. Frequent movement of juvenile rabbitfish between farming areas may further contribute to pathogen dissemination and connectivity among geographically separated cage‐farming systems. Rabbitfish may function as ecological bridge hosts, facilitating pathogen exchange between wild and cultured fish populations, linking wild fish populations with cultured stocks and facilitating the circulation and diversification of *S. iniae* within coastal aquaculture ecosystems. Genomic epidemiological approaches, such as multilocus sequence typing or whole‐genome sequencing, would provide valuable insights into transmission pathways and evolutionary relationships among rabbitfish isolates, other cultured species and surrounding wild fish populations [31, 32]. Long‐term monitoring of juvenile fish prior to cage stocking, combined with environmental sampling, would further clarify the relative importance of pathogen introduction from wild reservoirs versus persistence within farming systems. From a disease management perspective, surveillance efforts should be prioritised during warm‐water periods when outbreaks are most likely to occur. The use of healthy juveniles from verified sources, reduction of unnecessary fish transfers among farming areas, routine health monitoring and rapid removal of moribund fish may help limit pathogen transmission within marine cage networks. Because most isolates remained susceptible to TEs and β‐lactam antibiotics, antimicrobial treatments should be guided by susceptibility testing and integrated with broader biosecurity measures. In the longer term, the development of autogenous or commercial vaccines may provide a more sustainable approach to controlling streptococcosis in marine cage‐farmed rabbitfish.

In conclusion, the present findings establish *Streptococcus iniae* as a widely distributed cause of streptococcosis in marine cage‐farmed golden rabbitfish in Vietnam. The repeated detection of the bacterium across multiple provinces and over several years supports the sustained regional circulation of *S. iniae* within interconnected marine cage aquaculture systems. The combination of high pathogenicity, heterogeneous virulence gene profiles and variable antimicrobial susceptibility patterns underscores the adaptive potential of *S. iniae* populations in intensive cage‐farming environments. These findings provide new epidemiological insights into streptococcosis in siganid aquaculture and emphasise the importance of strengthened surveillance, biosecurity and responsible antimicrobial use to mitigate the emergence and spread of this pathogen.

## Author Contributions

**Truong Dinh Hoai**: conceptualisation, funding acquisition, investigation, methodology, project administration, data curation, formal analysis, resources, supervision, visualisation, writing – original draft, writing – review and editing. **Dang Thi Hoa, Nguyen Cong Thiet, Nguyen Thi Huong Giang and Doan Thi Nhinh**: data curation, formal analysis, methodology, visualisation. **Tran Thi Nang Thu and Kim Van Van**: conceptualisation, resources, supervision.

## Funding

This work was supported by the Académie de Recherche et d’Enseignement Supérieur (Grant NV2025‐09‐08TĐ).

## Ethics Statement

The authors confirm compliance with the journal’s ethical policies, as noted on the journal’s author guidelines page. Ethical approval for the challenge experiments was obtained from the Faculty of Fisheries, Vietnam National University of Agriculture Animal Care and Use Committee, FFVNUA‐ACUC, Approval Number 090825‐10‐KHCN‐FFVNUA.

## Conflicts of Interest

The authors declare no conflicts of interest.

## Supporting Information

Additional supporting information can be found online in the Supporting Information section.

## Supporting information


**Supporting Information** Figure S1. Sampling locations of marine cage farms affected by *Streptococcus iniae* outbreaks in golden rabbitfish in Vietnam. Figure S2. Final cumulative mortality of golden rabbitfish experimentally challenged with different doses of *Streptococcus iniae* at 14 days post‐challenge. Video S1. Representative clinical signs and abnormal swimming behaviour observed in naturally diseased golden rabbitfish affected by streptococcosis.

## Data Availability

The supporting information is in Figure S1, and Video S1 is available in Mendeley Data at https://doi.org/10.17632/8c7swfyy9z.1.
