# Supplementary material for: Emergence and Regional Circulation of Streptococcus iniae Associated With Streptococcosis in Marine Cage‐Farmed Golden Rabbitfish (Siganus guttatus)
Source: Transbound Emerg Dis. 2026 Jul 13;2026:2656593. doi: 10.1155/tbed/2656593 (PMC13365797; doi:10.1155/tbed/2656593)
Supplement: Supplementary file 1 — Supporting Information Figure S1. Sampling locations of marine cage farms affected by Streptococcus iniae outbreaks in golden rabbitfish in Vietnam. Figure S2. Final cumulative mortality of golden rabbitfish experimentally challenged with different doses of Streptococcus iniae at 14 days post‐challenge. Video S1. Representative clinical signs and abnormal swimming behaviour observed in naturally diseased golden rabbitfish affected by streptococcosis. [file TBED-2026-2656593-s001.pptx]

## Slide 1
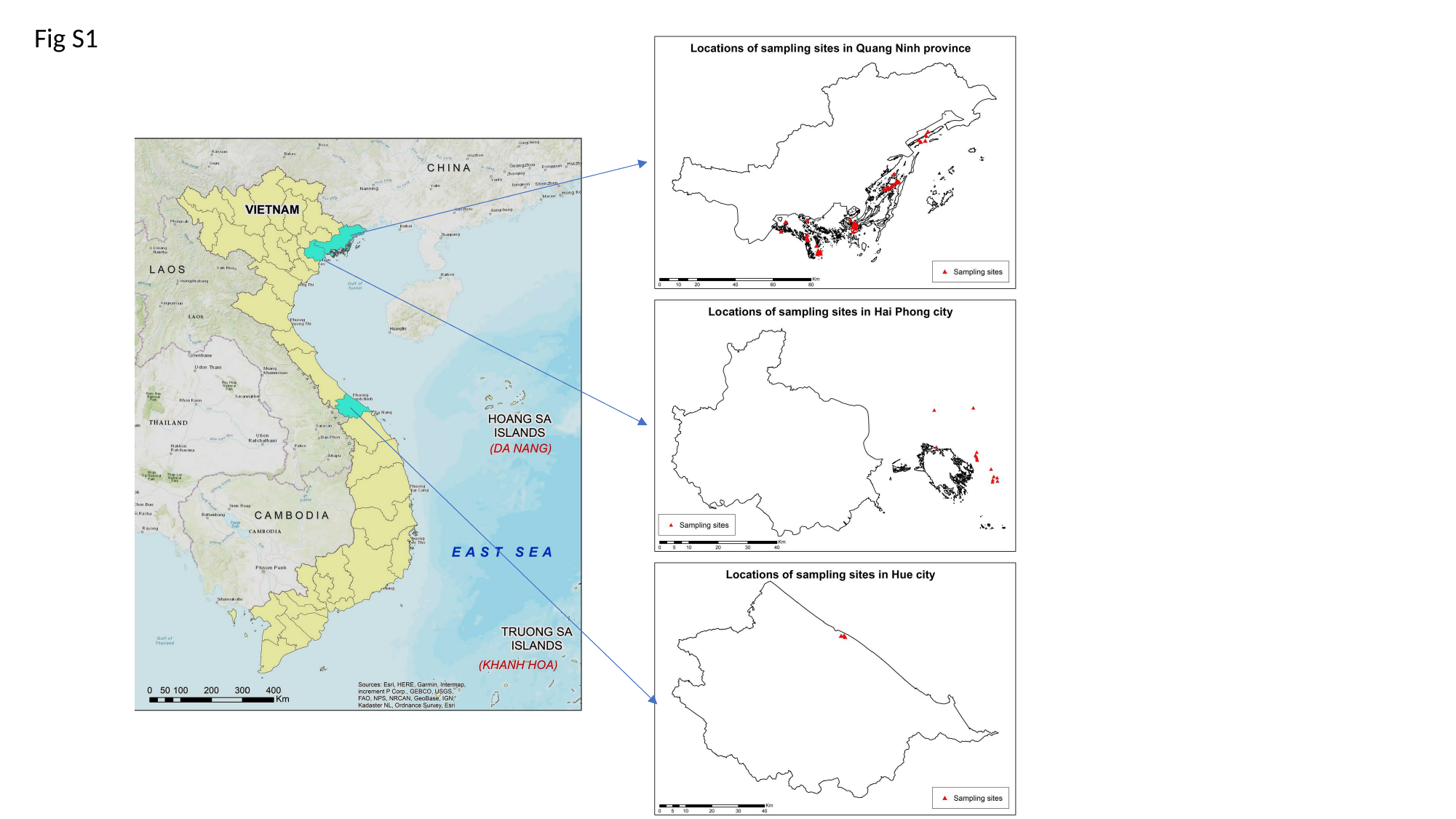

Fig S1

## Slide 2
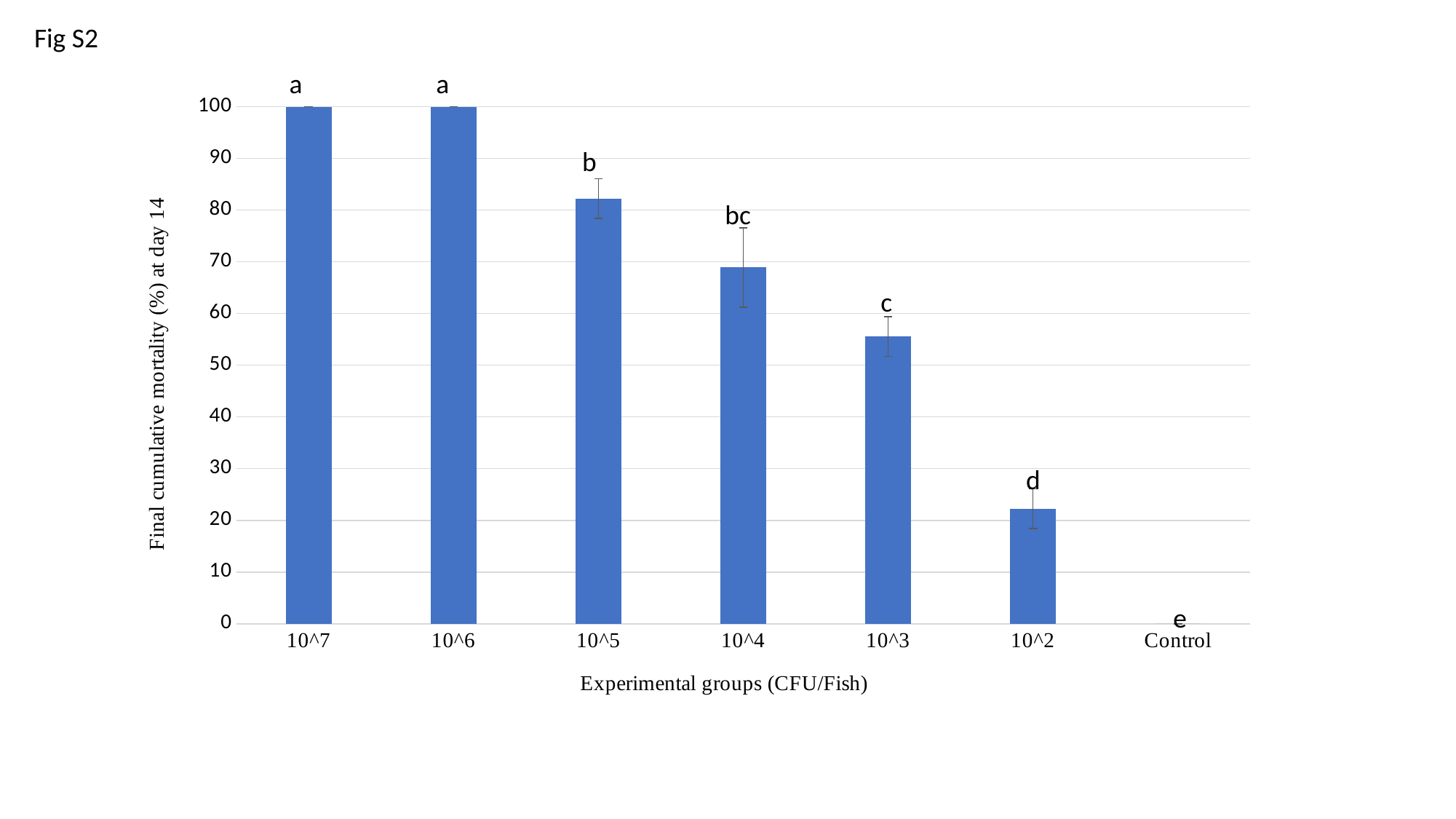

Fig S2
a
a
### Chart
| Category | |
|---|---|
| 10^7 | 100.0 |
| 10^6 | 100.0 |
| 10^5 | 82.22222222222223 |
| 10^4 | 68.88888888888889 |
| 10^3 | 55.555555555555564 |
| 10^2 | 22.222222222222225 |
| Control | 0.0 |b
bc
c
d
e

## Slide 3
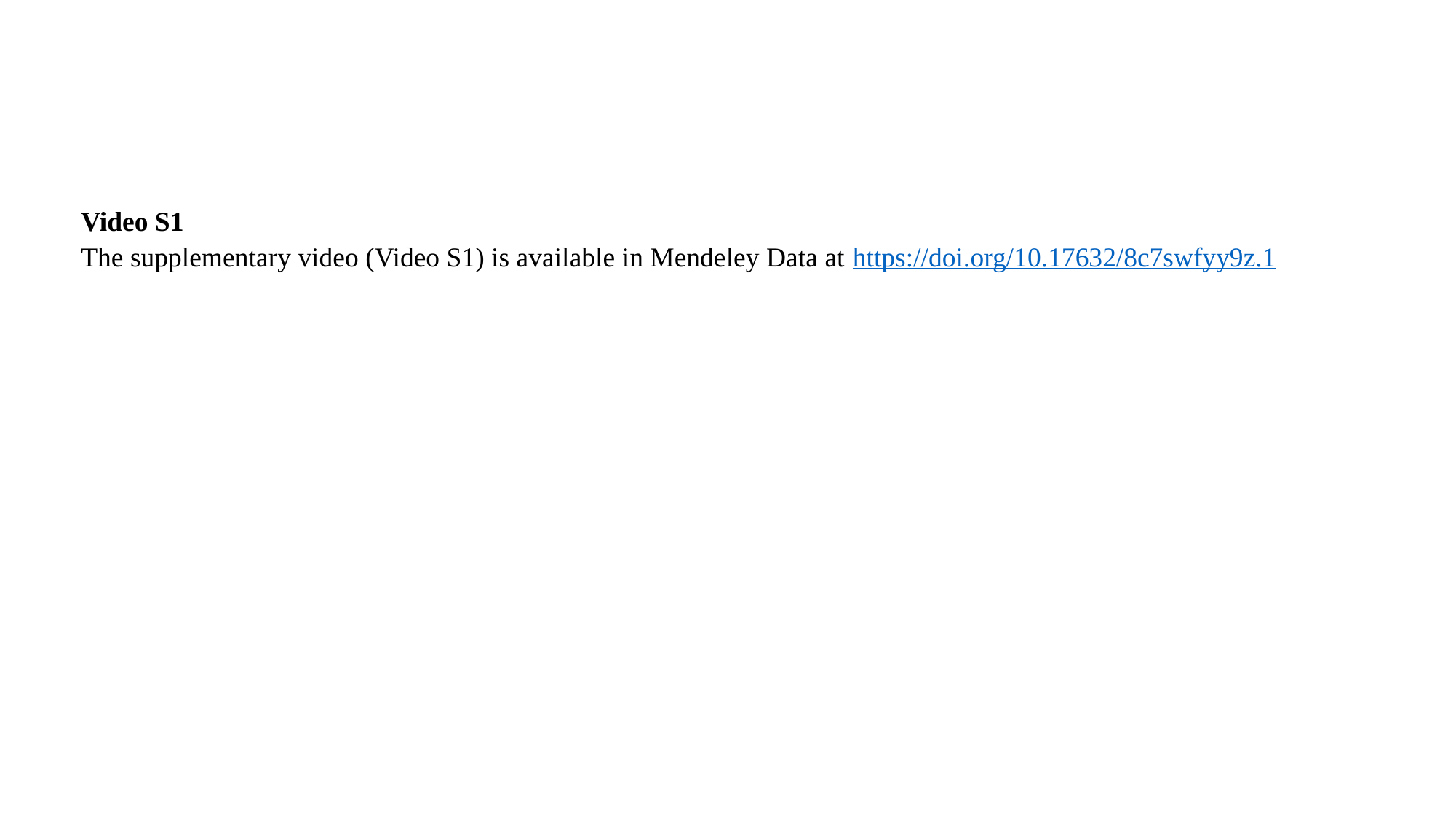

# Video S1 The supplementary video (Video S1) is available in Mendeley Data at https://doi.org/10.17632/8c7swfyy9z.1
